# Genetic evidence that SMAD2 is not required for gonadal tumor development in inhibin-deficient mice

**DOI:** 10.1186/1477-7827-8-69

**Published:** 2010-06-21

**Authors:** Saneal Rajanahally, Julio E Agno, Roopa L Nalam, Michael B Weinstein, Kate L Loveland, Martin M Matzuk, Qinglei Li

**Affiliations:** 1 Department of Pathology and Immunology, Baylor College of Medicine, Houston, Texas 77030, USA; 2 Department of Molecular and Cellular Biology, Baylor College of Medicine, Houston, Texas 77030, USA; 3 Department of Molecular and Human Genetics, Baylor College of Medicine, Houston, Texas 77030, USA; 4 Department of Biochemistry and Cell Biology, Rice University, Houston, Texas 77005, USA; 5 Department of Molecular Genetics and Division of Human Cancer Genetics, Ohio State University, Columbus, Ohio 43210, USA; 6 Departments of Biochemistry & Molecular Biology and Anatomy & Developmental Biology, Monash University, Clayton, Victoria 3800, Australia

## Abstract

**Background:**

Inhibin is a tumor-suppressor and activin antagonist. Inhibin-deficient mice develop gonadal tumors and a cachexia wasting syndrome due to enhanced activin signaling. Because activins signal through SMAD2 and SMAD3 in vitro and loss of SMAD3 attenuates ovarian tumor development in inhibin-deficient females, we sought to determine the role of SMAD2 in the development of ovarian tumors originating from the granulosa cell lineage.

**Methods:**

Using an inhibin α null mouse model and a conditional knockout strategy, double conditional knockout mice of Smad2 and inhibin alpha were generated in the current study. The survival rate and development of gonadal tumors and the accompanying cachexia wasting syndrome were monitored.

**Results:**

Nearly identical to the controls, the Smad2 and inhibin alpha double knockout mice succumbed to weight loss, aggressive tumor progression, and death. Furthermore, elevated activin levels and activin-induced pathologies in the liver and stomach characteristic of inhibin deficiency were also observed in these mice. Our results indicate that SMAD2 ablation does not protect inhibin-deficient females from the development of ovarian tumors or the cachexia wasting syndrome.

**Conclusions:**

SMAD2 is not required for mediating tumorigenic signals of activin in ovarian tumor development caused by loss of inhibin.

## Background

The transforming growth factor β (TGFβ) superfamily ligands including activins and inhibins play integral roles in a wide variety of developmental processes [1-3]. Inhibins are α and β subunit heterodimers (inhibin A: α, βA; inhibin B: α, βB) that oppose activin signaling by antagonizing activin receptors (ACVRs), whereas activins are homodimers (activin A, βA: βA; activin B, βB: βB) or heterodimers (activin AB, βA: βB) of the β subunits [4-6]. Activin signal transduction is initiated when the ligand binds to its type 2 serine/threonine kinase receptor which in turn phosphorylates the type 1 receptor [7-11]. The type 1 receptor then phosphorylates and activates receptor-regulated SMADs (R-SMADs; SMAD2 and SMAD3), which subsequently form complexes with the common SMAD, SMAD4. The R-SMADs/SMAD4 can translocate into the nucleus to regulate gene expression via recruitment of specific transcription factors, activators, and repressors [12-15].

Activins and inhibins are expressed in ovarian granulosa cells and were first described for their roles in FSH regulation [16,17]. However, subsequent studies demonstrated the involvement of these ligands in multiple developmental and pathological events including carcinogenesis [18-20]. Inhibin is a tumor suppressor [21], as inhibin α (*Inha*) null mice develop gonadal sex cord-stromal tumors originating from the granulosa/Sertoli cell lineages [21], presumably due to the loss of activin antagonism. The tumors secrete an excessive amount of activins that signal through activin receptor type 2 (ACVR2) in the stomach and liver, leading to a cachexia wasting syndrome and pathological changes in these organs (depletion of parietal cells in the glandular stomach and hepatocellular death in the liver) [22,23]. Lethality in *Inha *null mice is primarily caused by the cachexia wasting syndrome characterized by weight loss, lethargy, and anemia [24]. Although the mechanisms of tumorigenesis in *Inha *null mice are not fully understood, activin, FSH, and estradiol may play pivotal roles in the development of gonadal tumors [25-28]. As absence of an α subunit precludes α:β dimer assembly, activin is highly elevated in *Inha *null mice due to the ability of the β subunits to only form β:β activin dimers [24]. While activin-deficient mice die after birth due to craniofacial defects [9], accumulating evidence suggest that activins play important roles in gonadal tumor development in inhibin-deficient mice. Expression of the activin βA subunit is elevated in the gonads of inhibin-deficient mice [29]. Moreover, tumorigenesis is attenuated in inhibin-deficient mice that transgenically express follistatin, an activin antagonist [30,31]. More recently, we demonstrated that administration of a chimeric ACVR2 ectodomain (ActRII-mFc), a known activin antagonist, delayed gonadal tumorigenesis in inhibin-deficient mice [32].

To dissect the activin downstream signaling components during ovarian tumorigenesis, we previously generated *Inha/Smad3 *double knockout mice in which females are substantially, but not completely, protected from the development of ovarian tumors and the accompanying cachexia syndrome [28]. Since SMAD2 and SMAD3 are activin signal-transducers *in vitro *and the gonadal somatic cells (granulosa cells and Sertoli cells) from which inhibin-deficient tumors are derived express both SMADs, we hypothesized that SMAD2 may partially compensate for the loss of SMAD3 in mediating ovarian activin signals in the *Inha/Smad3 *double knockout females. To circumvent the embryonic lethality of *Smad2 *ubiquitous knockout [33-35], we conditionally deleted *Smad2 *in ovarian granulosa cells null for *Inha *to determine the role of SMAD2 in gonadal tumor development.

## Methods

### Generation of *Inha/Smad2 *conditional knockout mice

**Figure 1:**
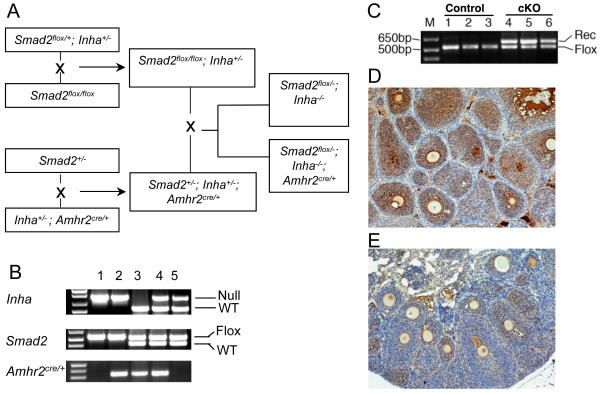
**Generation of *Inha/Smad2 *cKO mice**. (A) *Smad2*^*flox/+*^; *Inha*^*+/- *^mice were mated with *Smad2*^*flox/flox *^mice to produce *Smad2*^flox/flox^; *Inha*^*+/- *^mice, and *Smad2*^*+/- *^mice were mated with *Inha*^*+/-*^; *Amhr2*^*cre/+ *^mice to generate *Smad2*^*+/-*^; *Inha*^*+/-*^; *Amhr2*^*cre/+ *^mice. These mice were then crossed to produce *Smad2*^flox/-^; *Inha*^*-/-*^; *Amhr2*^*cre/+ *^mice (experimental group) and *Smad2*^flox/-^; *Inha*^*-/- *^mice (control group). The generation of the *Inha *null, *Smad2 *null and *Smad2 *floxed alleles is described in the *Materials and Methods*. (B) Genotyping of mice using PCR analysis. Representative PCR images are shown with corresponding genotypes listed below: 1. *Inhα*^*-/-*^; *Smad2*^*flox/-*^; *Amhr2*^*+/+ *^; 2. *Inhα*^*-/-*^; *Smad2*^*flox/-*^; *Amhr2*^cre/+ ^; 3. *Inhα*^*+/+*^; *Smad2*^*flox/+*^; *Amhr2*^*cre/+ *^; 4. *Inhα*^*+/-*^; *Smad2*^*flox/+*^; *Amhr2*^*cre/+*^; 5. *Inhα^*+/-*^; Smad2^*flox/+*^; Amhr2^*+/+*^. (C) Recombination of Smad2 floxed allele in Inhα^*-/-*^; Smad2^*flox/-*^; Amhr2^*cre/+ *^*tumors. Note that the recombined (Rec) allele of *Smad2 *can be detected only in the tumor tissues of the *Inha/Smad2 *cKO mice but not in the controls where the Cre recombinase is not expressed. (D and E) Immunohistochemical analysis of SMAD2 protein expression in *Smad2*^flox/-^; *Inha*^*-/-*^; *Amhr2*^*+/+ *^(D) and *Smad2*^*flox/-*^; *Inha*^*-/-*^; *Amhr2*^*Cre/+*^(E) mice. Both panels are representative images. Note the dramatic reduction of SMAD2 protein expression in the conditional knockout mice vs. controls (100 × magnification).

Mice used in this study were maintained on a mixed C57BL/6/129S6/SvEv background and manipulated according to the NIH *Guide for the Care and Use of Laboratory Animals*. Generation of the *Inha *null mice and the *Smad2 *null allele was described previously [21,36]. The *Smad2 *conditional allele was constructed by flanking exons 9 and 10 with two *loxP *sites using the Cre-LoxP system as previously documented [37,38]. The *Amhr2*^*cre/+ *^mice were produced via insertion of a Cre-Neo cassette into the fifth exon of the anti-Mullerian hormone receptor type 2 (*Amhr2*) locus [39]. Generation of the *Smad2*^flox/-^; *Inha*^*-/-*^; *Amhr2*^cre/+ ^mice (experimental group) and *Smad2*^*flox/-*^; Inha^*-/- *^mice (control group) is depicted in Figure 1.

### Genotyping analysis

Genotyping of the mice was performed by PCR using genomic tail DNA. Table 1 lists the primer sequences utilized in the PCR assays. The annealing temperatures for *Inha*, *Amhr2*^cre/+^, and *Smad2 *were 61°C, 62°C, and 60°C, respectively. The resultant PCR products were separated and visualized on 1% agarose gels.

**Table 1:** Primer sequences for genotyping PCR

Gene	Primer sequence (5'-3')	
	Forward	Reverse
*Inha *WT	cct ggg tgg cgc agg ata tgg	ggt ctc ctg cgg ctt tgc gc
*Inha *null	cct ggg tgg cgc agg ata tgg	gga tat gcc ctt gac tat aat g
*Amhr2cre*	cgc att gtc tga gta ggt gt	gaa acg cag ctc ggc cag c
*Smad2 *flox	tac ttg ggg caa tct ttt cg	gtc act ccc tga acc tga ag
*Smad2 *null	gct gag tgc cta agt gat agt gca	tct tct ttt tcc ccg ctg g
*Smad2 *Rec	gag ctg cgc aga cct tgt tac	gtc act ccc tga acc tga ag

Rec, recombined floxed allele

### Measurement of body weight and generation of survival curve

Body weights of animals were measured and recorded weekly from ages 4-26 weeks, and the mice were closely monitored for the development of the cachexia wasting syndrome (i.e., weight loss, kyphoscoliosis, and lethargy) [24]. Mice were sacrificed when their body weights fell below 15 grams or when other severe cachexia symptoms developed as described elsewhere [24,40]. All mice were sacrificed at the end of 26 weeks for a final analysis. To determine the potential effect of conditional deletion of *Smad2 *on ovarian tumor development at early stages, the *Inha/Smad2 *cKO mice were also examined at 4 to 9 weeks of age.

### Histological analysis

Mice were anesthetized by isoflurane inhalation at the time of sacrifice. A small portion of the tails were cut and stored at -70 °C for subsequent genotype verification. Ovaries, stomachs, and livers were removed from the mice and fixed in 10% (vol/vol) neutral buffered formalin overnight. The fixed samples were washed with 70% ethanol prior to paraffin embedding. Ovaries were sectioned and stained with periodic acid-Schiff (PAS)-hematoxylin, whereas livers and stomachs were processed for hematoxylin and eosin (HE) staining. All staining procedures were conducted in the Pathology Core Services Facility at Baylor College of Medicine using standard protocols.

### Immunohistochemistry

Expression of SMAD2 in *Smad2*^flox/-^; *Inha*^*-/-*^; *Amhr2*^cre/+ ^and *Smad2*^*flox/-*^; *Inha*^*-/- *^mice was determined by immunohistochemistry. Briefly, ovaries from 4-week-old mice were fixed in formalin and serially sectioned (5 μm). Antigen retrieval was performed by boiling the sections in 10 mM citrate buffer (pH 6.0). The sections were then blocked using 3% BSA/10% serum in PBS, and incubated with SMAD2 primary antibody (1:100 dilution; Cell Signaling). Subsequent procedures were performed using ABC and DAB kits (Vector Lab). The sections were counterstained with hematoxylin and mounted with Permount.

### Activin A analysis

Blood samples were collected from anesthetized mice by cardiac puncture upon sacrifice, placed in serum separator tubes (Becton Dickinson, Franklin Lakes, NJ, USA), and allowed to clot at room temperature. Serum was then isolated from the blood samples by centrifugation and stored at -20°C until assayed. Total serum activin A levels were measured using a specific ELISA [41] according to the manufacturer's instructions (Oxford Bio-Innovations, Oxfordshire, UK) with modifications [42]. The average intraplate coefficient of variation (CV) was 7.4% and the interplate CV was 10.8% (*n *= 2 plates). The limit of detection was 0.01 ng/ml.

### Statistical analyses

Differences among groups (average ovary weight, liver weight, and serum activin A levels) were assessed using one-way ANOVA followed by a Kruskal-Wallis post-hoc test. The survival curve was analyzed using a logrank test. For all analyses, significance was defined at *P *< 0.05. Data are reported as mean standard error of the mean (SEM).

## Results

### Generation of *Smad2*^*flox/-*^; *Inha*^*-/-*^; *Amhr2*^*cre/+ *^mice

To understand the roles of SMAD2 in ovarian tumor development in inhibin-deficient mice, we took advantage of a conditional knockout strategy to disrupt the *Smad2 *gene in mouse ovarian granulosa cells. To overcome the embryonic lethality phenotype resulting from *Smad2 *ubiquitous deletion, a *Smad2 *floxed allele was generated by flanking exons 9 and 10 of the *Smad2 *gene with 2 *loxP *sites [37]. The *Amhr2*^*cre/+ *^knock-in mouse line validated in our previous studies to delete genes expressed in ovarian granulosa cells [38,43-47] was utilized. Figure 1A depicts the breeding scheme used to generate the control (*Smad2*^flox/-^; *Inha*^*-/-*^) and experimental *Smad2*^flox/-^; *Inha*^*-/-*^; *Amhr2*^*cre/+ *^(*Inha/Smad2 *cKO) female mice. Representative genotype analyses are presented in Figure 1B. We previously demonstrated that the *Smad2 *floxed allele can be recombined and deleted in mouse granulosa cells in *Smad2 *cKO mice [38]. As a further support that inhibin-deficient tumors originate from granulosa cells, the *Smad2 *recombined allele was readily detectable in the tumor tissues of *Smad2*^flox/-^; *Inha*^*-/-*^; *Amhr2*^*cre/+ *^mice, but not in the controls lacking the Cre-recombinase (Figure 1C). Moreover, immunostaining revealed a dramatic reduction of SMAD2 protein levels in the granulosa cells of *Smad2*^flox/-^; *Inha*^*-/-*^; *Amhr2*^*cre/+ *^mice vs. controls (Figure 1D and 1E).

### Conditional knockout of *Smad2 *in inhibin-deficient mice does not alter lethality

**Figure 2:**
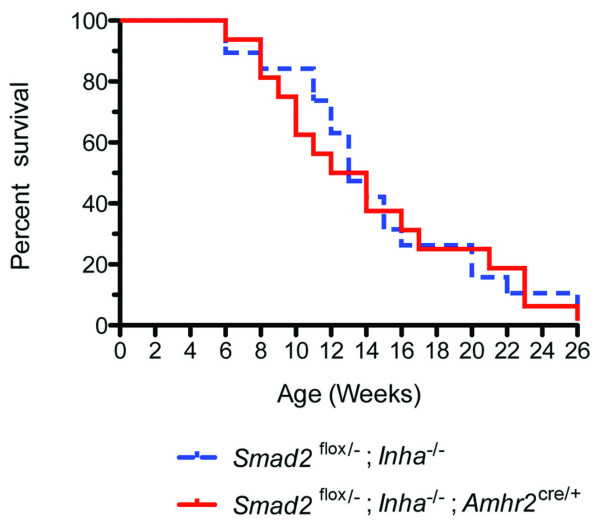
**Survival curve of *Smad2*^*flox/-*^; *Inha*^*-/- *^and *Smad2*^*flox/-*^; *Inha*^*-/-*^; *Amhr2*^*Cre/+ *^mice**. The survival of the *Smad2*^*flox/-*^; *Inha*^*-/- *^(*n *= 19; control group) and *Smad2*^*flox/-*^; *Inha*^*-/-*^; *Amhr2*^*Cre/+ *^(*n *= 16; experimental group) mice were recorded weekly during 4 to 26 weeks. The survival curve was generated and analyzed using a Mantel-Cox test (GraphPad Software, GraphPad Prism version 5.0 b for MacOS X). Statistical significance was not found between the two groups [χ^2 ^(1, N = 35) = 0.051, *P *= 0.82].

To evaluate the overall effects of *Smad2 *conditional deletion on the life span of inhibin-deficient mice, we generated and analyzed the survival curves of the *Smad2*^*flox/-*^; *Inha*^*-/- *^controls (*n *= 19) and the *Smad2*^*flox/-*^; *Inha*^*-/-*^; *Amhr2*^*cre/+ *^(*n *= 16) experimental mice (Figure 2). The median survival was 13 weeks for both *Smad2*^*flox/-*^; *Inha*^*-/- *^and *Smad2*^*flox/-*^;*Inha *^*-/-*^; *Amhr2*^*cre/+ *^females. Statistical analysis indicated that *Smad2 *deficiency did not alter the lifespan of *Inha *null mice (*P *> 0.05).

### Development of ovarian tumors and cachexia wasting syndrome in *Smad2*^*flox/-*^; *Inha*^*-/-*^; *Amhr2*^*cre/+ *^mice

**Figure 3:**
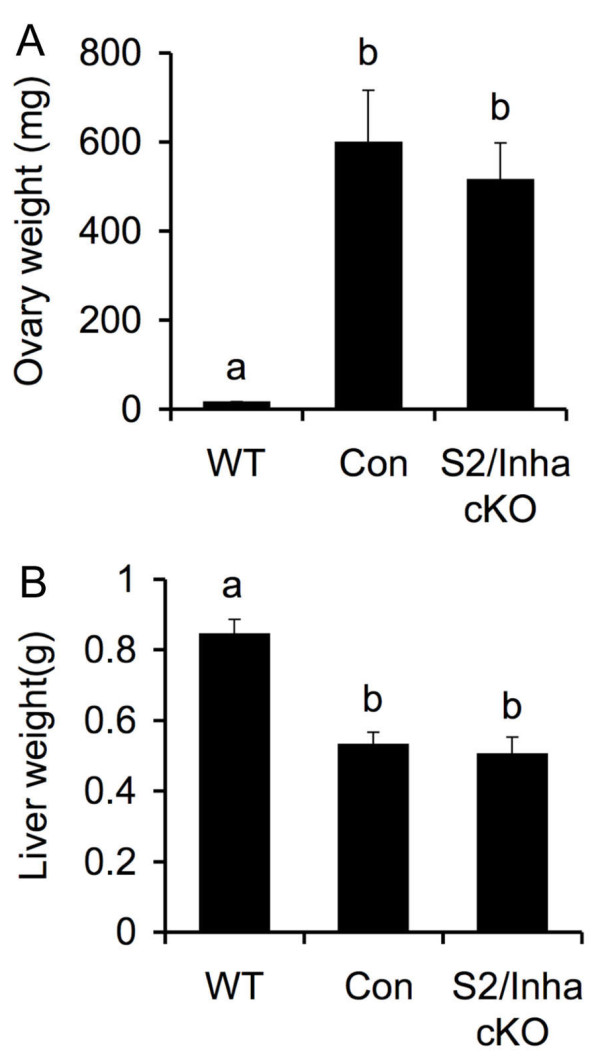
**Ovary and liver weights of WT, *Smad2*^*flox/-*^; *Inha*^*-/- *^control (Con) and *Smad2*^*flox/-*^; *Inha*^*-/-*^; *Amhr2*^*cre/+*^(*S2/Inha *cKO) experimental mice**. Note the dramatic alteration of the weights of the ovary and liver of the *Smad2*^*flox/-*^; *Inha*^*-/- *^(6-26 wk; *n *= 11) and *Smad2*^*flox/-*^; *Inha*^*-/-*^; *Amhr*^*cre/+*^(6-23 wk; *n *= 5) mice compared to adult WT mice (12 wk; *n *= 5) due to tumor development. However, no differences in the ovary and liver weights were observed between the control and *S2/Inha *cKO mice. All data are shown as mean ± SEM, and bars without a common letter are significantly different at *P *< 0.01.

An early sign of tumor development in *Inha *null mice is severe body weight loss, a prominent feature of the cachexia wasting syndrome. To determine if *Smad2 *deficiency affects the development and progression of the cachexia syndrome, the body weights of *Smad2*^*flox/-*^; *Inha*^*-/- *^and *Smad2*^*flox/-*^; *Inha*^*-/-*^; *Amhr2*^*cre/+ *^mice were measured weekly. The results showed that *Smad2*^*flox/-*^; *Inha*^*-/-*^; *Amhr2*^*cre/+ *^mice suffered from weight loss similar to that observed in *Smad2*^*flox/-*^; *Inha*^*-/- *^mice (data not shown). Because the cachexia syndrome is also characterized by distinct activin-induced pathological changes in the stomach and liver [22,23], these organs were collected and examined along with the gonads. The ovary and liver weights of the wild type (WT), *Smad2*^*flox/-*^; *Inha*^*-/-*^, and *Smad2*^*flox/-*^; *Inha*^*-/-*^; *Amhr2*^*cre/+ *^mice were measured. Despite the marked changes of the weights of ovary and liver in both *Smad2*^*flox/-*^; *Inha *^*-/- *^(*n *= 11) and *Smad2*^*flox/-*^; *Inha*^*-/-*^; *Amhr2*^*cre/+ *^mice (*n *= 5) compared to WT mice (*n *= 5; *P *< 0.01), no significant differences in these parameters were found between the *Smad2*^*flox/-*^; *Inha*^*-/-*^; *Amhr2*^*cre/+ *^mice and the *Smad2*^*flox/-*^; *Inha*^*-/- *^controls at a similar stage of tumor progression (*P *> 0.05; Figure 3).

**Figure 4:**
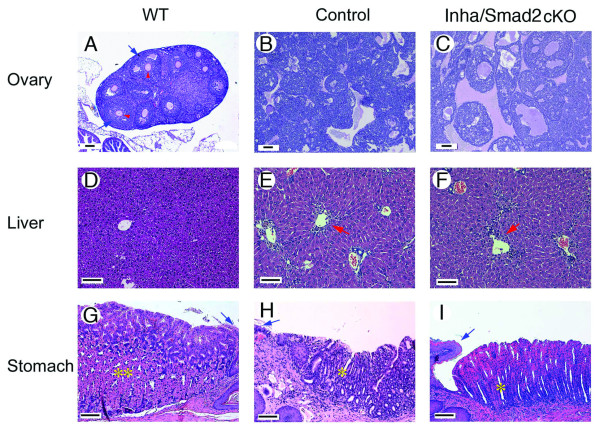
**Histological analyses of ovary, liver, and stomach from WT, control, and experimental female mice**. (A) Ovarian histology of an 8 wk old WT mouse. The ovary contains follicles at various developmental stages. Arrows indicate granulosa cells and arrowheads denote oocytes surrounded by a magenta-colored zona pellucida. (B and C) Ovarian histology of a 26 wk old *Smad2*^*flox/-*^; *Inha*^*-/- *^mouse (B) and a 23 wk old *Smad2*^*flox/-*^; *Inha*^*-/-*^; *Amhr2*^*cre/+*^mouse (C), respectively. Tumors from both genotypes were bilateral, large, hemorrhagic, and histologically indistinguishable. (D-F) Histology of livers from a 12 wk old WT mouse (D), a 15 wk old *Smad2*^*flox/-*^; *Inha*^*-/- *^mouse (E), and an 8 wk old *Smad2*^*flox/-*^; *Inha*^*-/-*^; *Amhr2*^*cre/+*^mouse (F). Hepatocellular death around the central vein and lymphocyte infiltration are present in the livers of both the *Smad2*^*flox/-*^; *Inha*^*-/- *^control and *Smad2*^*flox/-*^; *Inha*^*-/-*^; *Amhr2*^*cre/+*^mice (arrows). (G-I) Glandular stomachs from a 12 wk old WT mouse (G), a 26 wk old *Smad2*^*flox/-*^; *Inha*^*-/- *^mouse (H), and a 14 wk old *Smad2*^*flox/-*^; *Inha*^*-/-*^; *Amhr2*^*cre/+*^mouse (I). Note the depletion of parietal cells in both the control and experimental mice (single asterisk) in comparison with the WT mouse (the large and eosinophilic cells; double asterisks). The junction ridge between the squamous epithelium of the forestomach and the glandular stomach is indicated by blue arrows. Scale bars = 100 μm.

Histological analyses were performed on the ovaries and livers of WT, *Smad2*^*flox/-*^; *Inha*^*-/-*^, and *Smad2*^*flox/-*^; *Inha*^*-/-*^; *Amhr2*^*cre/+*^mice. We first examined the histology of ovaries and livers of the *Smad2*^*flox/-*^; *Inha*^*-/-*^; *Amhr2*^*cre/+*^mice and the *Smad2*^*flox/-*^; *Inha*^*-/- *^mice at the same advanced tumor stage when severe cachexia was observed. In the absence of inhibins, ovarian tumors were grossly hemorrhagic and contained blood-filled cysts irrespective of the status of SMAD2 (Figure 4B and 4C). Microscopic analysis of the livers demonstrated hepatocellular death and lymphocyte infiltration around the central vein (Figure 4E and 4F), another activin-induced pathological effect [22,24]. Furthermore, glandular stomachs of both the control and experimental groups were characterized by depletion of eosinophilic parietal cells and glandular atrophy (Figure 4H and 4I). The observed liver and stomach pathologies are consistent with those of cachectic inhibin-deficient mice [24].

Next, to uncover potential effect of *Smad2 *deletion on ovarian tumor development at an early stage, we examined the tumor status in the *Smad2*^*flox/-*^; *Inha*^*-/-*^; *Amhr2*^*cre/+ *^mice at various time points between 4 and 9 weeks of age, since inhibin-deficient mice can develop tumors as early as 4 weeks. At 4 weeks of age, significant differences were not found in either the cachexia syndrome associated parameters (ovary and liver weights) or tumor histology between the controls (*n *= 3) and the *Smad2*^*flox/-*^; *Inha*^*-/-*^; *Amhr2*^*cre/+ *^(*n *= 3) mice (*P *> 0.05). Similar results were obtained when comparisons were performed at both 6 weeks (*n *= 3 for each group) and 8-9 weeks of age (*n *= 3 for each group) (data not shown). Thus, conditional deletion of *Smad2 *does not delay ovarian tumor development and the progression of the cachexia wasting syndrome in inhibin-deficient mice.

### Activin levels

Serum activins are elevated in the inhibin-deficient mice due to the excessive production of the β subunits from gonadal tumors [24]. Thus, activin levels correlate with the tumor status in mice lacking inhibin. The superphysiological level of activins is the primary cause of the cachexia syndrome [22,24]. Since both *Smad2*^*flox/-*^; *Inha*^*-/- *^and *Smad2*^*flox/-*^; *Inha*^*-/-*^; *Amhr2*^*cre/+ *^mice displayed the severe cachexia syndrome, and ovarian tumors in these mice were histologically indistinguishable, we proposed that conditional deletion of *Smad2 *does not alter the production of activins, an indicator of tumor status in mice lacking inhibins. To test this hypothesis, we measured activin A levels in *Smad2*^*flox/-*^; *Inha*^*-/-*^; *Amhr2*^*cre/+ *^mice and the corresponding control mice at the advanced tumor stage, and found that levels of activin A were similarly elevated in both *Smad2*^*flox/-*^; *Inha*^*-/- *^control (*n *= 11; 54.1 ± 8.2 ng/ml) and *Smad2*^*flox/-*^; *Inha*^*-/-*^; *Amhr2*^*cre/+ *^experimental females (*n *= 5; 47.0 ± 6.7 ng/ml) (*P *> 0.05) in comparison to WT females (*n *= 7; 0.1 ± 0.0 ng/ml).

## Discussion

The aim of the current study was to define the role of SMAD2 in the development of ovarian tumors and activin-induced cancer cachexia syndrome. We demonstrated that conditional deletion of SMAD2 did not prevent the inhibin-deficient females from ovarian tumorigenesis and death; all *Inha/Smad2 *cKO mice developed sex cord-stromal tumors resembling those observed in *Inha *null mice. Furthermore, *Inha/Smad2 *cKO mice suffered from the cancer cachexia syndrome, as evidenced by the severe weight loss and pathological lesions in the stomach and liver (i.e., mucosal atrophy with depletion of parietal cells in the glandular stomach and hepatocellular necrosis around the central vein) [24]. These results indicate that SMAD2 is not required for transducing superphysiological activin signals in the context of gonadal tumor development due to loss of inhibin.

Activins play complex roles in carcinogenesis. In several extragonadal tissues, activin A has been reported to be an anti-tumorigenic factor. For example, activin prevents cell proliferation in breast cancer through SMAD2/3-dependent regulation of cell cycle arrest genes [48]. Similarly, activin A acts as a tumor suppressor in neuroblastoma cells via inhibition of angiogenesis, a key feature of tumorigenesis. Inhibition of endothelial cell proliferation can be achieved by active forms of SMAD2 and SMAD3, suggesting this inhibitory effect is SMAD2/3 dependent [49]. Moreover, activin A has also been reported to prevent the proliferation of tumor cells derived from the prostate and gall bladder [50,51].

Despite the above anti-tumorigenic effects of activins in extragonadal tissues, activins promote tumor development in the gonads [28,52]. The tumorigenic roles of activins have been suggested by the *Inha *knockout mouse model [21], and the inhibin-deficient mouse model has been exploited to gain a deep understanding of the activin signaling pathway in gonadal tumor development. The *Inha/Smad3 *double knockout mice generated in our previous study highlights the importance of activins in gonadal tumorigenesis [28]. Since deletion of SMAD3 only delays ovarian tumor development in the *Inha *null mice [28], we were interested in determining the potential involvement of SMAD2 in mediating the potentiated activin signaling in ovarian tumors lacking inhibins.

SMAD2 and SMAD3 are functionally distinct proteins. Structural differences at the MH1 domain exist between SMAD2 and SMAD3. The extra amino acids (encoded by exon 3) in the SMAD2 MH1 domain prevents its direct binding to DNA, and specific transcription factors are required for SMAD2-DNA binding [53-55]. In contrast, SMAD3 has direct DNA-binding ability [56,57]. Additionally, SMAD3-SMAD4 signaling-dependent genes outnumber SMAD2-SMAD4 dependent genes by more than 4-fold, as identified in Hep3B cells in a recent microarray experiment [58]. Finally, distinct signaling outcomes have been identified in developing mouse Sertoli cells linked with developmentally regulated, differential use of SMAD2 and SMAD3 [59]. Despite these distinctive aspects, SMAD2 and SMAD3 share more than 90% identity in their amino acid sequences [60], and functional redundancy between these two proteins has been demonstrated in the ovary [58,38].

**Figure 5:**
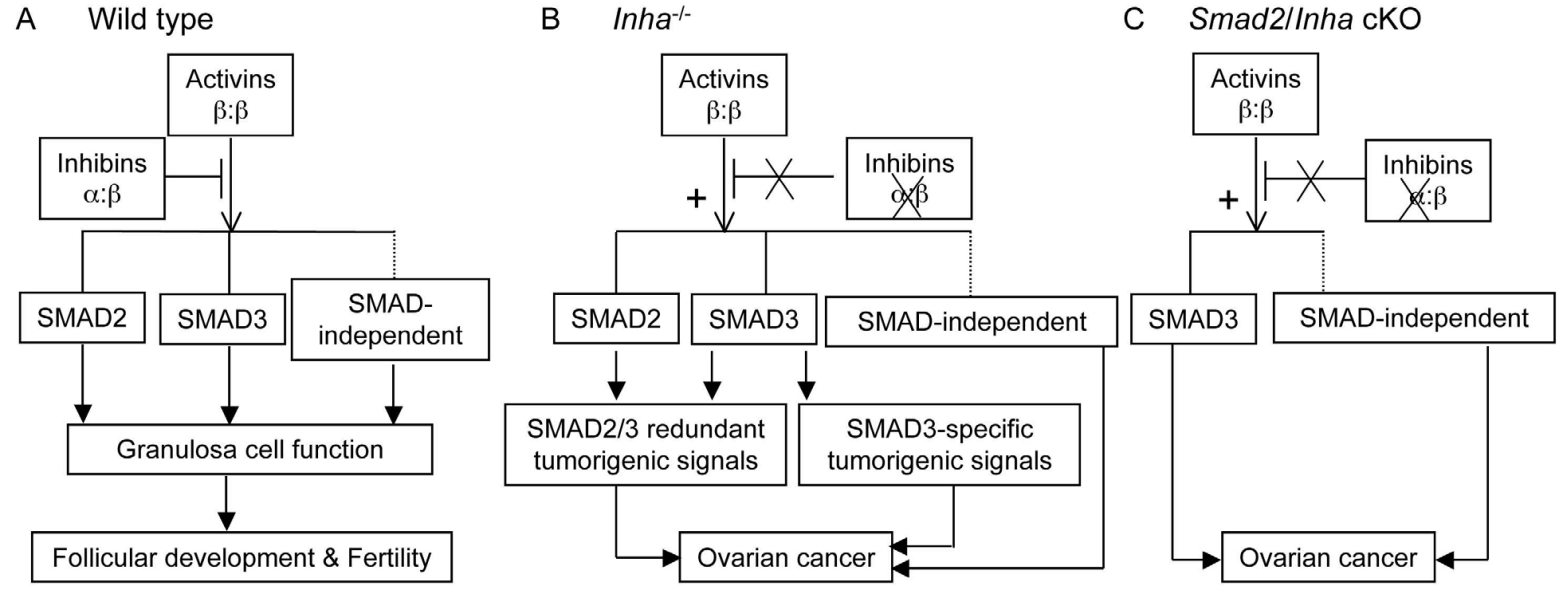
**Hypothetical working model for SMAD2/3 signaling in mediating gonadal tumorigenesis in inhibin-deficient female mice**. **(A) **In the WT ovary, the signaling of activins is finely tuned by inhibins, which is important in the maintenance of normal granulosa cell function, follicular development, and fertility. **(B) **In the absence of inhibins, activin signaling is potentiated with increased production of activins by the gonads and tumors due to the loss of antagonism by inhibins. Superphysiological levels of activins can signal through both SMAD2 and SMAD3 in the ovary. As demonstrated by the *Inha/Smad3 *double knockout mouse model [28], ovarian tumor development is attenuated in *Inha *null mice lacking SMAD3, implying that the function of SMAD3 is not fully compensated by SMAD2. Complementarily, the *Inha/Smad2 *cKO mouse model generated in the current study suggests that SMAD3 can potentially mediate essential tumorigenic signals of activins in the *Inha *null mice **(C)**. However, our model does not rule out the potential involvement of SMAD-independent signaling (dotted lines) in inhibin-deficient ovarian tumor development or the possibility that SMAD2 may not be involved in gonadal tumor development.

Our current findings, in combination with our previous results, indicate that SMAD2 and SMAD3 may function redundantly to mediate gonadal tumorigenesis in inhibin-deficient mice. In the case of conditional deletion of *Smad2*, SMAD3 compensates for the deficiency of SMAD2 and transduces essential signals contributing to ovarian tumor development; consequently, tumorigenesis is not altered. On the other hand, loss of SMAD3 in the *Inha *null mice attenuates but does not prevent ovarian tumor development, suggesting that SMAD2 may partially compensate for the loss of SMAD3. However, our model does not rule out the potential involvement of SMAD-independent signaling in inhibin-deficient ovarian tumor development or the possibility that SMAD2 may not be involved in gonadal tumor development (See Figure 5 for details). It will be interesting to further explore if the contrasting role of activins in gonadal versus extragonadal tissues is linked to the differential impingement of downstream SMAD2 and/or SMAD3 transducers. Furthermore, the potential crosstalk between SMAD-dependent and SMAD-independent signaling pathways in inhibin-deficient tumor development awaits further investigation.

## Conclusions

SMAD2 is not required for mediating tumorigenic signals of activin in ovarian tumor development caused by loss of inhibin.

## Abbreviations

Inha: inhibin α; cKO: conditional knockout; TGFβ: transforming growth factor β; ACVR: activin receptor; WT: wild type.

## Competing interests

The authors declare that they have no competing interests.

## Authors' contributions

SR performed the experiments and drafted the manuscript. JEA and RLN helped SR to perform genotyping and immunohistochemistry analyses. MBW generated *Smad2 *mutant mice. KLL performed activin assays and revised manuscript. MMM and QL designed and supervised this study and revised the manuscript. All authors read and approved the final manuscript.
